# Using a length-weight relationship based on wild lumpfish (*Cyclopterus lumpus* L.) for estimation of body condition of lumpfish in salmon cages

**DOI:** 10.1371/journal.pone.0310924

**Published:** 2024-11-12

**Authors:** Sandra Ljósá Østerø, Jessica Di Toro, Eydna í Homrum, Esbern J. Patursson, Kirstin Eliasen

**Affiliations:** 1 Firum, Hvalvík, Faroe Islands; 2 Institute of Aquaculture, University of Stirling, Stirling, Scotland, United Kingdom; 3 Faroe Marine Research Institute, Tórshavn, Faroe Islands; 4 Hiddenfjord, Sandavágur, Faroe Islands; Mansoura University, EGYPT

## Abstract

The use of lumpfish (*Cyclopterus lumpus* L.) as cleaner fish in Atlantic salmon aquaculture has emerged as a promising solution to combat the issue of sea lice infestation, as they have proven effective under the right conditions. The aquaculture industry, however, is facing challenges in ensuring the welfare and survival of lumpfish in salmon cages. Part of the welfare monitoring of lumpfish is the estimation of body condition. Previous research on standard length-weight relationships for lumpfish has primarily relied on the growth patterns of lumpfish in salmon cages, which might not align with the natural growth patterns of the fish. In this study, we argue for another perspective: using the standard weight of wild lumpfish to estimate the body condition of lumpfish in salmon cages. We assert that this approach aligns more closely with the natural growth pattern of the fish and may offer a more accurate representation of their condition. Our findings show significant differences in growth patterns between wild lumpfish, which show positive allometric growth, and those in salmon cages, which show slightly negative allometric growth. The findings underscore the importance of using appropriate length-weight relationships for lumpfish in aquaculture to ensure an accurate assessment of their body condition.

## Introduction

The lumpfish (*Cyclopterus lumpus* L.), along with other cleaner fish species, play an important role in combating sea lice infestations on farmed Atlantic salmon. Sea lice pose a substantial challenge to the salmon farming industry [1], leading to the use of cleaner fish as a biological control measure to mitigate this issue. Under the right conditions, lumpfish have proven effective in reducing sea lice infestations on farmed Atlantic salmon [2].

Unlike wrasse, lumpfish are native to the Faroe Islands, making them the only cleaner fish species used in the local salmon production. The first lumpfish were introduced to salmon cages in the Faroe Islands in 2014. The number of lumpfish used as cleaner fish increased gradually to 2.2 million in 2020 but then declined to 0.6 million in 2023 (Faroese Food and Veterinary Authority, personal communication) due to high mortalities and varying effects.

To improve the survival and welfare of lumpfish in salmon cages, it is important to understand the relations between husbandry practices and lumpfish health and welfare. Lumpfish are a relatively new species in aquaculture and remain largely non-domesticated, with most broodstock being wild-caught. To improve welfare and reduce mortality, there is a need for species-specific welfare indicators to establish good practices for the management of lumpfish in salmon cages. Various manuals and guidelines on standardized welfare indicators have been published e.g. [3–5].

Part of the welfare monitoring of lumpfish in salmon cages is the estimation of body condition. A recent study observed that 33–60% of lumpfish were assesed as being underweight or emaciated in salmon cages [6], suggesting that the nutritional requirements of lumpfish in salmon cages are not fully met. There is therefore a need for a correct species-adapted method for estimating the body condition of lumpfish in salmon cages.

To our knowledge, all previous work on lumpfish body condition in salmon cages has been based on standard weight calculations derived from fitted regressions including weight and length measurements of lumpfish in salmon cages [5, 7–9]. One such standard weight [5] has been widely used in various other publications [3, 6, 10, 11].

Here, we argue for another perspective: using the standard weight of wild lumpfish to estimate the body condition of lumpfish in salmon cages. We assert that this approach aligns more closely with the natural growth pattern of the fish and may offer a more accurate representation of their body condition.

## Materials and methods

### Wild lumpfish

**Fig 1:**
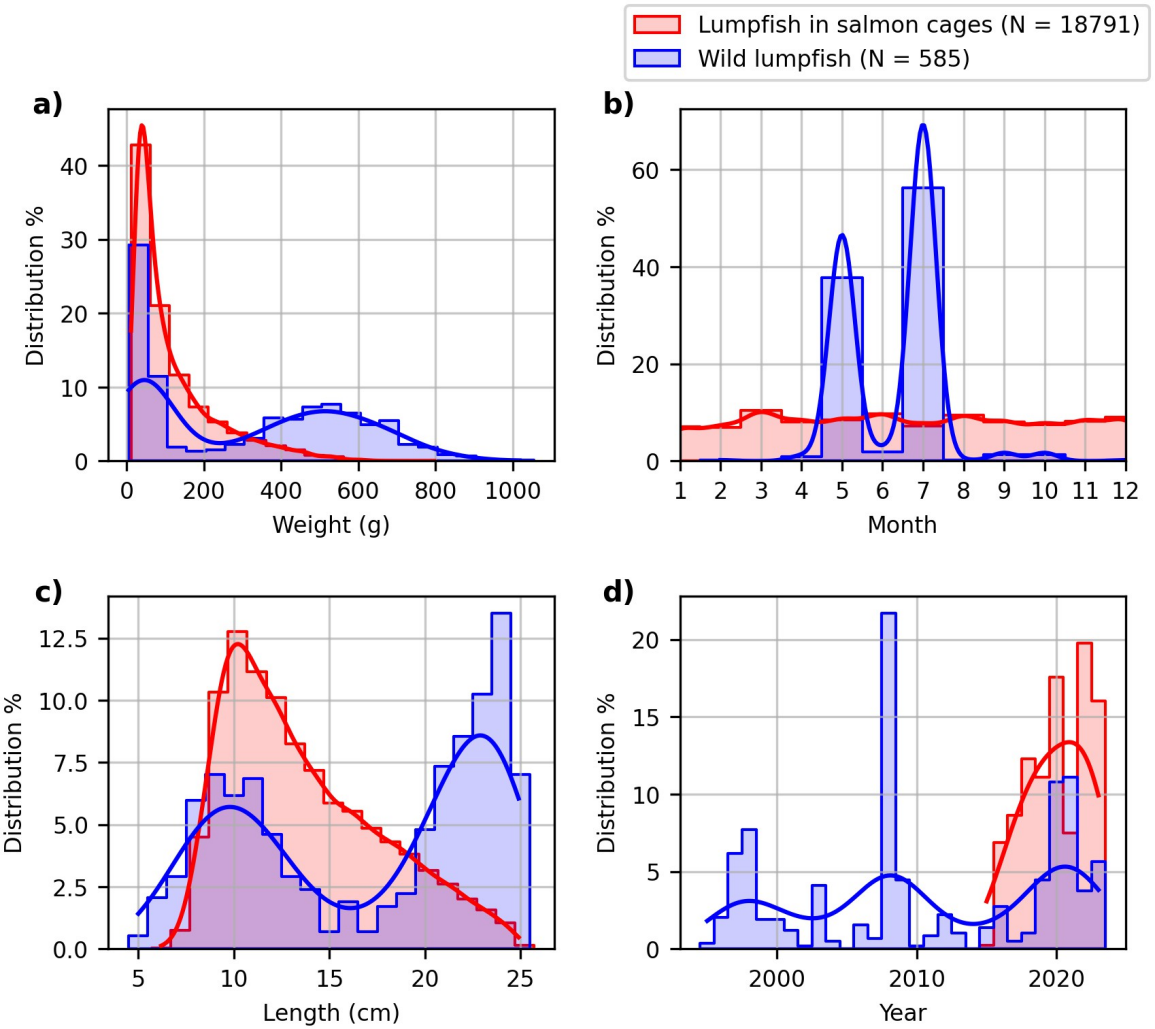
Data overview. Overview of lumpfish datasets. Blue represents wild lumpfish and red represents lumpfish in salmon cages. **a)** Weight distribution, **b)** monthly distribution of sampling **c)** length distribution, **d)** yearly distribution of sampling.

Data on the length and weight of wild lumpfish were from annual pelagic research surveys conducted by the Faroe Marine Research Institute (Havstovan) from 1995 to 2023. To avoid sexually mature fish, only lumpfish below 25 centimetres in total length were included in this study, as males reach maturity at approximately 25–35 centimetres and females at approximately 35–45 centimetres [12]. The original data included 1628 wild lumpfish. After excluding the potentially sexually mature fish (>25 centimetres) there were 585 lumpfish left in the dataset. An overview of the dataset is provided in Fig 1. The wild lumpfish are caught within a geographical area bounded by latitudes 60.19°N and 71.18°N, and longitudes -13.94°W and 3.94°W.

### Lumpfish in salmon cages

Data on the length and weight of lumpfish in salmon cages were from lumpfish monitoring programs at eight different Atlantic salmon farms in the Faroe Islands. The data was from 283 sampling days from December 2015 to September 2023. The weight of the lumpfish was measured to the nearest gram and the length was measured to the nearest millimetre from the mouth to the end of the caudal fin (total length). Lumpfish with a fin score of 3, indicating severely damaged caudal fins in the scoring system developed by Eliasen et al. [7], were omitted from the analysis. After filtering, there were 18,791 lumpfish in the dataset. An overview of the dataset is provided in Fig 1.

### Length-weight relationship

Length-weight relationships of fishes are of the power type *W* = *a* ⋅ *L*^*b*^ [13], where *W* is the predicted weight to a certain length (*L*). If *b* = 3, the growth of the fish is isometric, if *b* < 3 the growth is negatively allometric, i.e., the fish grows faster in length than weight, and if *b* > 3 the growth is positively allometric, i.e., the fish grows faster in weight than length [14]. To calculate the coefficients *a* and *b*, linear regression was used on the log-transformed equation: log_10_(*W*_*s*_) = log_10_(*a*) + *b* ⋅ log_10_(*L*). The regression models show the expected standard weight (*W*_*s*_), representing the best-fitting regression. In this study, the length-weight relationship of wild lumpfish was used as the expected standard weight (*W*_*s*_) for lumpfish in salmon cages. The body condition of individual fish was assessed by calculating the relative weight (*W*_*r*_), defined as the ratio of the actual weight (*W*) to the expected weight (*W*_*s*_), expressed as a percentage using the equation: *W*_*r*_ = (*W*/*W*_*s*_) ⋅ 100 [15]. Based on the following criteria from Rabadan et al. [5], the fish were then categorized to reflect their body condition: good condition (*W*_*r*_ > 90%), underweight (*W*_*r*_ = 75–90%) and emaciated (*W*_*r*_ < 75%).

### Statistical analysis

All data and statistical analyses were conducted using the open-source software Python 3.9.7, provided by the Python Software Foundation. The statsmodels module [16] version 0.13.5 was used for regression analyses. Using ordinary least squares (OLS) regression, linear regression analyses were performed on log-transformed length and weight data to derive the length-weight regressions. To test for an effect of groups (wild lumpfish vs. lumpfish in salmon cages, May-July samples vs. August-April samples) on length, linear regressions with weight as the response variable and interaction between group and length as predictor variables were conducted.

## Results and discussion

Fitting the log-transformed equation to the length and weight data of the wild lumpfish resulted in the length-weight relationship

$$\begin{array}{c} {\text{log}}_{10}\left(W_{s}\right)=-1.477+3.094\cdot {\text{log}}_{10}\left(L\right)\;\left(R^{2}=0.979\right), \end{array}$$

where *W*_*s*_ is the expected weight (g) and L is the total length (cm). The intercept represents the logarithm of the coefficient *a* in the power equation *W* = *a* ⋅ *L*^*b*^. As *b* > 3, the growth of the wild lumpfish is positively allometric.

Fitting the log-transformed equation to the data of the lumpfish in salmon cages from this study resulted in the length-weight relationship

$$\begin{array}{c} {\text{log}}_{10}\left(W_{s}\right)=-1.383+2.945\cdot {\text{log}}_{10}\left(L\right)\;\left(R^{2}=0.964\right), \end{array}$$

meaning a slightly negative allometric growth of the fish. An interaction analysis showed a statistically significant difference in the length-weight relationship between wild lumpfish and lumpfish in salmon cages (*p* < 0.001). This indicates that the growth patterns, as represented by the relationship between length and weight, significantly differ between the two groups.

The majority of wild lumpfish (96%) were collected between May and July. To determine if seasonal variations influenced the growth pattern differences observed between wild lumpfish and those in salmon cages, an interaction analysis was conducted. The analysis compared the growth patterns of lumpfish in salmon cages during the May-July period with those from the remaining months. The analysis revealed no significant difference (p = 0.068) in the length-weight relationship of lumpfish in salmon cages between the May-July period and the August-April period, suggesting that growth patterns of lumpfish in salmon cages were consistent across seasons. A summary of the regression models is presented in Table 1.

**Table 1:** Summary of length-weight regression models for wild lumpfish and lumpfish in salmon cages.

Regression model	Size range (cm)	N	a ± SE	b ± SE	*R* ^2^
Wild lumpfish	5.0—24.9 (TL)	585	-1.477 ± 0.023	3.094 ± 0.019	0.979
Farmed lumpfish	6.2—24.9 (TL)	18791	-1.383 ± 0.005	2.945 ± 0.004	0.964
S4. Post-deployment [5]	7.0—16 (TL)	355	-0.957	2.559 ± 0.058	0.847
Total length *L*_*T*_ [17]	5.0—25.0 (SL)	29669	-0.961	2.502	

Models are based on log-transformed data: *log*_10_(*W*_*s*_) = *a* + *b* ⋅ *log*_10_(*L*), where *W*_*s*_ is weight (g) and *L* is length (cm). TL: Total length, SL: Standard length.

**Fig 2:**
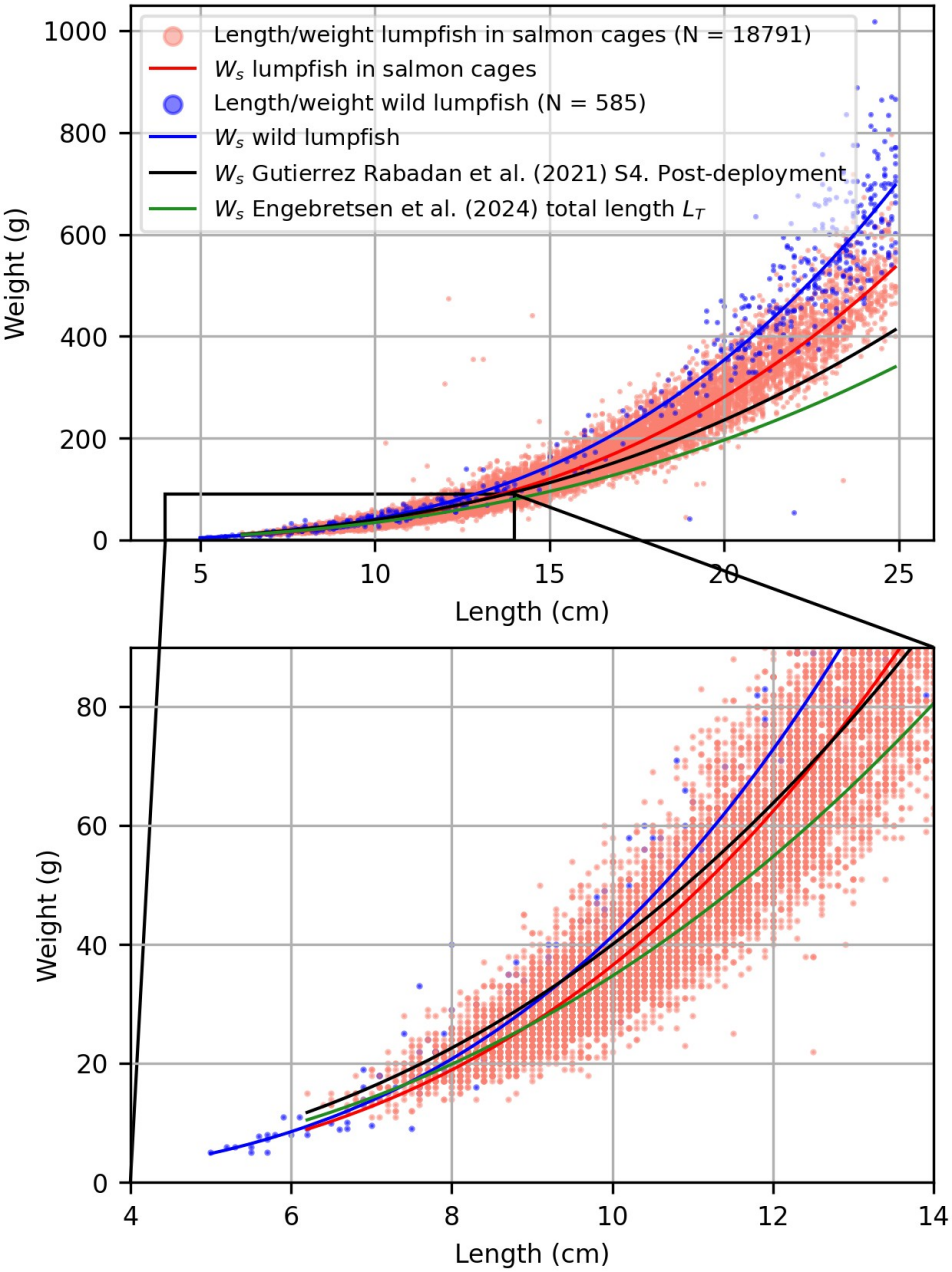
Length-weight relationships. Length-weight relationships (*W*_*s*_) of wild lumpfish in this study (blue), lumpfish in salmon cages in this study (red), S4. Post-deployment model [5] (black) and total length (*L*_*T*_) model [17] (green). Red scatter points show the actual length and weight of lumpfish in salmon cages in this study (N = 18791), and blue scatter points show the length and weight of wild lumpfish in this study (N = 585). Lengths are total length (cm).

To provide context, these results are compared with previously published length-weight relationships for lumpfish in salmon cages [5, 17] (Table 1 and Fig 2). In the study by Gutierrez Rabadan et al. [5] they found the length-weight relationship of lumpfish to vary significantly between life stages, and therefore developed 4 models; S1. Larve, S2. Pre-deployment, S3. Pre-deployment and S4. Post-deployment. The post-deployment model is being widely used as a standard weight for lumpfish in salmon cages in various other publications, e.g. [3, 6, 10, 11]. It thus forms the basis for comparison with the wild lumpfish model in this study. Since the upper limit of the weight range (g) of the S4. Post-deployment model is not disclosed, it is assumed that the regression model applies to lumpfish throughout their growth phase post-deployment in salmon cages.

As they found an estimated *b* of 2.502, the recent work by Engebretsen et al. [17] showed a similar estimated *b* as the post-deployment model by Gutierrez Rabadan et al. [5], where they found a *b* of 2.559. The study by Engebretsen et al. [17] was based on almost 30,000 lumpfish from Norwegian salmon farms. As their lumpfish ranged from 5 to 25 centimetres in standard length, it is similar to the data in the present study. However, with an estimated *b* of 2.945, our historical dataset of >18, 000 lumpfish shows a very different result. Wild lumpfish have served as a standard reference for evaluating the weight-to-length ratio of lumpfish in salmon cages since the onset of the lumpfish welfare monitoring program in the Faroe Islands in 2015 (personal communication). Throughout the years of the historical dataset, Faroese farmers have thus aimed to reach weights similar to that of wild lumpfish, which might explain the different result or part of it.

**Fig 3:**
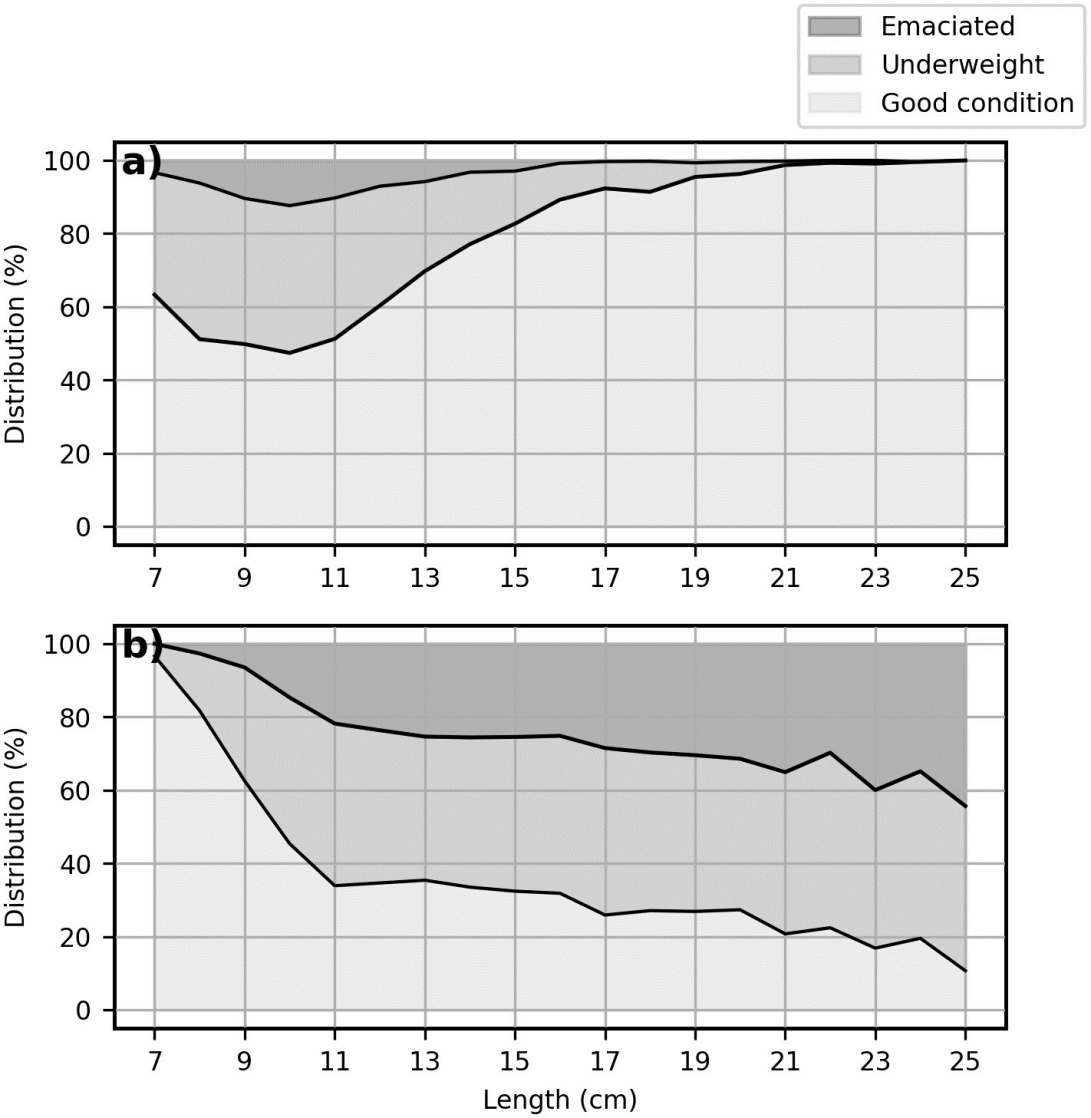
Proportional distribution of body condition categories. Variation in the proportion of lumpfish (N = 18791) that are in good condition (Wr>90%), underweight (Wr = 75–90%), and emaciated (Wr<75%). **a)** Based on the S4. Post-deployment regression [5] and **b)** wild lumpfish regression in this study. The limits are as defined by Gutierrez Rabadan et al. [5].

The difference in growth patterns between wild lumpfish and lumpfish in salmon cages holds significant implications for assessing lumpfish body condition in aquaculture settings. The S4. Post-deployment regression model [5] shows different characteristics compared to the regression models derived for both wild lumpfish and the lumpfish in salmon cages in this study. Specifically, the S4. Post-deployment model features a higher intercept (*a*) and a lower exponent (*b*). As a result, the model expects lumpfish to be heavier than the wild lumpfish up to a length of ∼9 cm, and heavier than the lumpfish in salmon cages in this study up to a length of ∼12 cm. On the contrary, the model expects that beyond these thresholds, lumpfish will weigh less than both wild lumpfish and lumpfish in salmon cages in this study (Fig 2). The consequence of this difference is further shown in Fig 3a and 3b. According to the S4. Post-deployment model [5], approximately 40% of the smallest fish are categorized as underweight or emaciated. In contrast, the wild lumpfish model categorizes nearly all of these lumpfish as being in good condition. The wild lumpfish model aligns well with observations from the Faroe Islands, where lumpfish body condition generally is good in hatcheries and at deployment, but frequently is observed to decline after some time in the salmon cages (personal communication). In contrast, the S4. Post-deployment model [5] shows a pattern, indicating that the smallest lumpfish are in the worst condition, with body condition improving as they grow (Fig 3a).

In total, approximately 64% of the > 18, 000 lumpfish in the historical dataset in this study were categorized as underweight or emaciated according to the length-weight regression derived from wild lumpfish data, along with the *W*_*r*_ limits from Rabadan et al. [5]. In contrast, only 28% of the > 18, 000 lumpfish were categorized as underweight or emaciated when using the S4. Post-deployment regression model [5].

## Conclusion

Previous research on length-weight relationships for lumpfish in salmon cages has primarily relied on data from lumpfish in salmon cages. However, it is important to note that the majority of lumpfish broodstock used in aquaculture is wild-caught. Using length-weight relationships derived from wild lumpfish may better align with the natural growth patterns of the fish and provide a more accurate representation of their condition. It is important to recognise, that a negative allometric growth pattern of lumpfish in salmon cages should not be perceived as the expected standard weight, but possibly as an indicator of sub-optimal growth conditions within the salmon cages. Using length-weight regressions fitted to lumpfish in salmon cages can lead to an overestimation of the actual body condition of the lumpfish. This overestimation may hinder the ability of farmers to detect underweight lumpfish, which is a crucial aspect in the welfare management of lumpfish.

## Future work

To better understand annual variations in growth patterns of wild lumpfish, future research should prioritize the collection and analysis of length and weight data of wild lumpfish across different seasons. More data on wild lumpfish would also provide a more robust baseline for developing accurate length-weight relationships for lumpfish. Additionally, further studies are needed to validate the applicability of the body condition limits established by Rabadan et al. [5] for the wild lumpfish model.

## Supporting information

S1 File Wild lumpfish data. (CSV)

S2 File Lumpfish in salmon cages data. (CSV)

S1 Raw images (PDF)
