# Supplementary figures and images for: Using a length-weight relationship based on wild lumpfish (Cyclopterus lumpus L.) for estimation of body condition of lumpfish in salmon cages
Source: PLoS One. 2024 Nov 12;19(11):e0310924. doi: 10.1371/journal.pone.0310924 (PMC11556749; doi:10.1371/journal.pone.0310924)

Wild lumpfish (N = 585)  
Lumpfish in salmon cages (N = 18791)

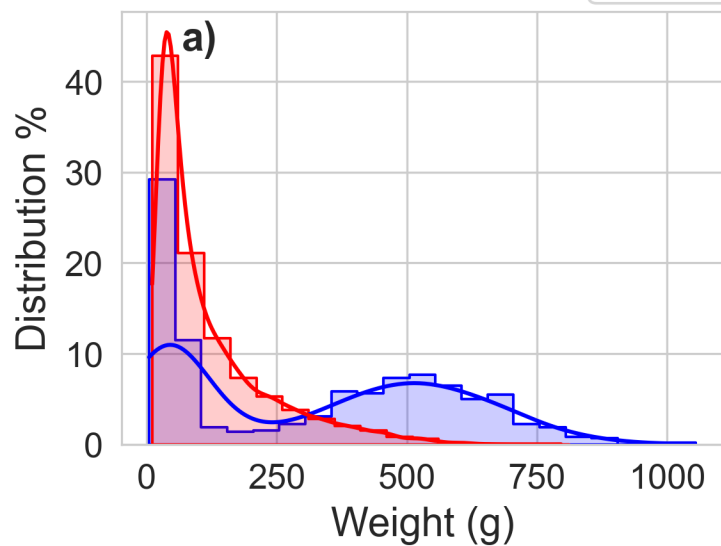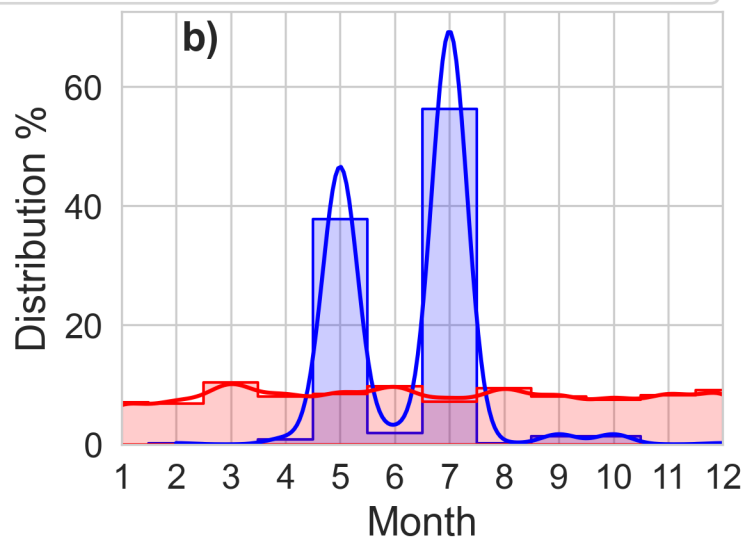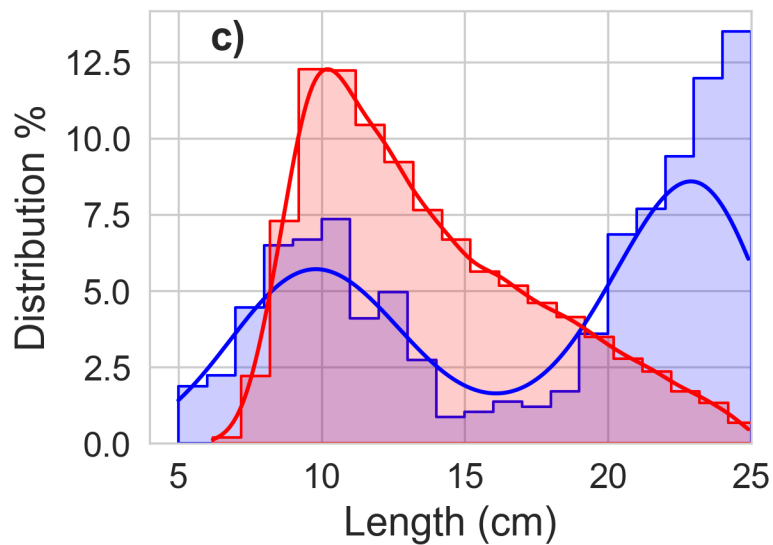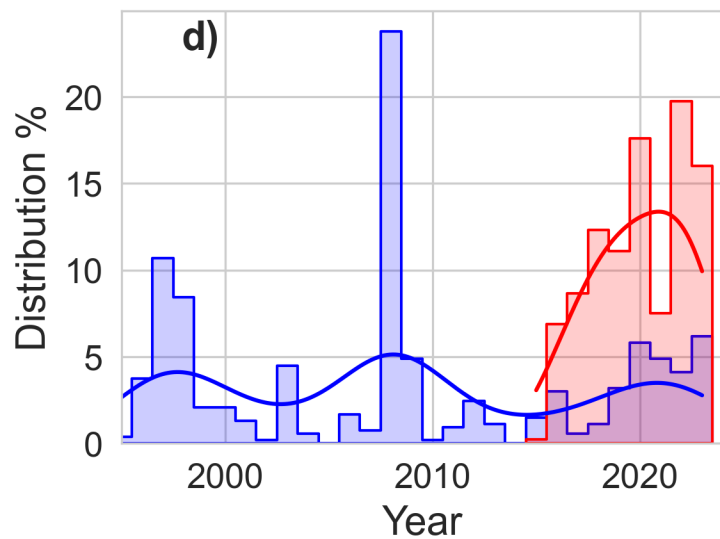

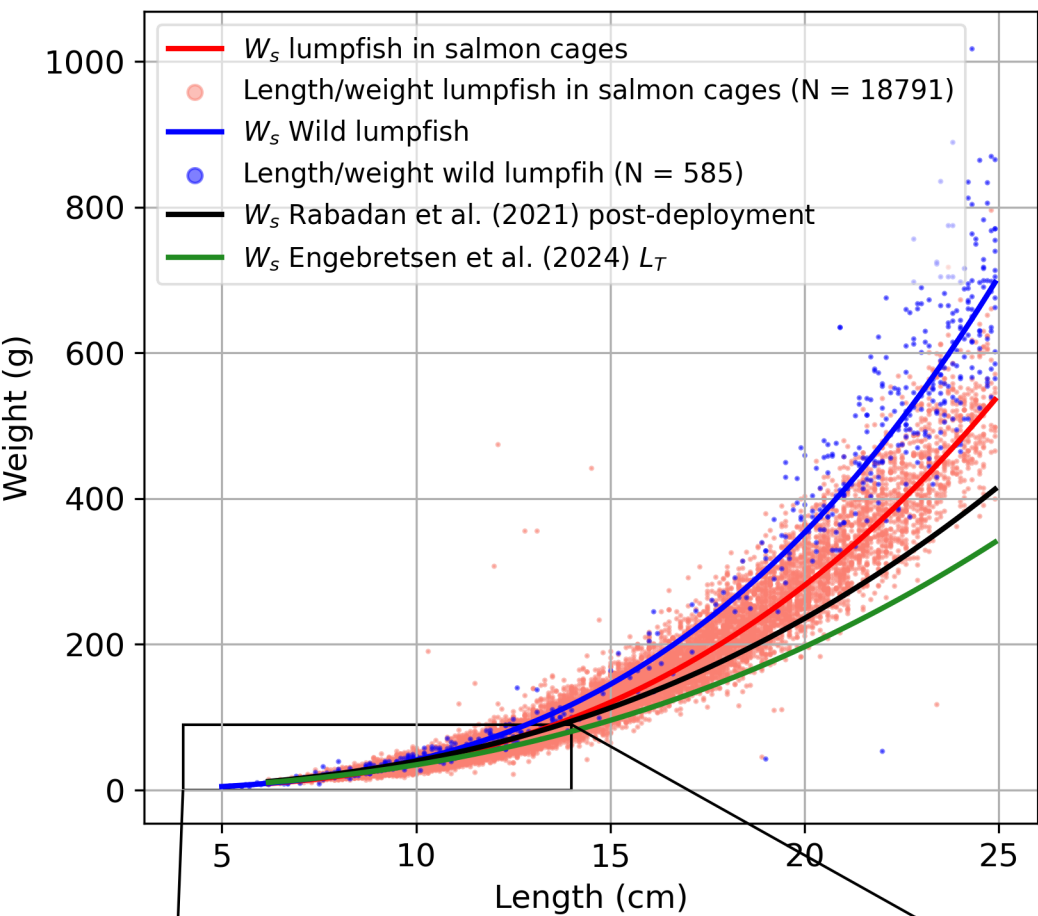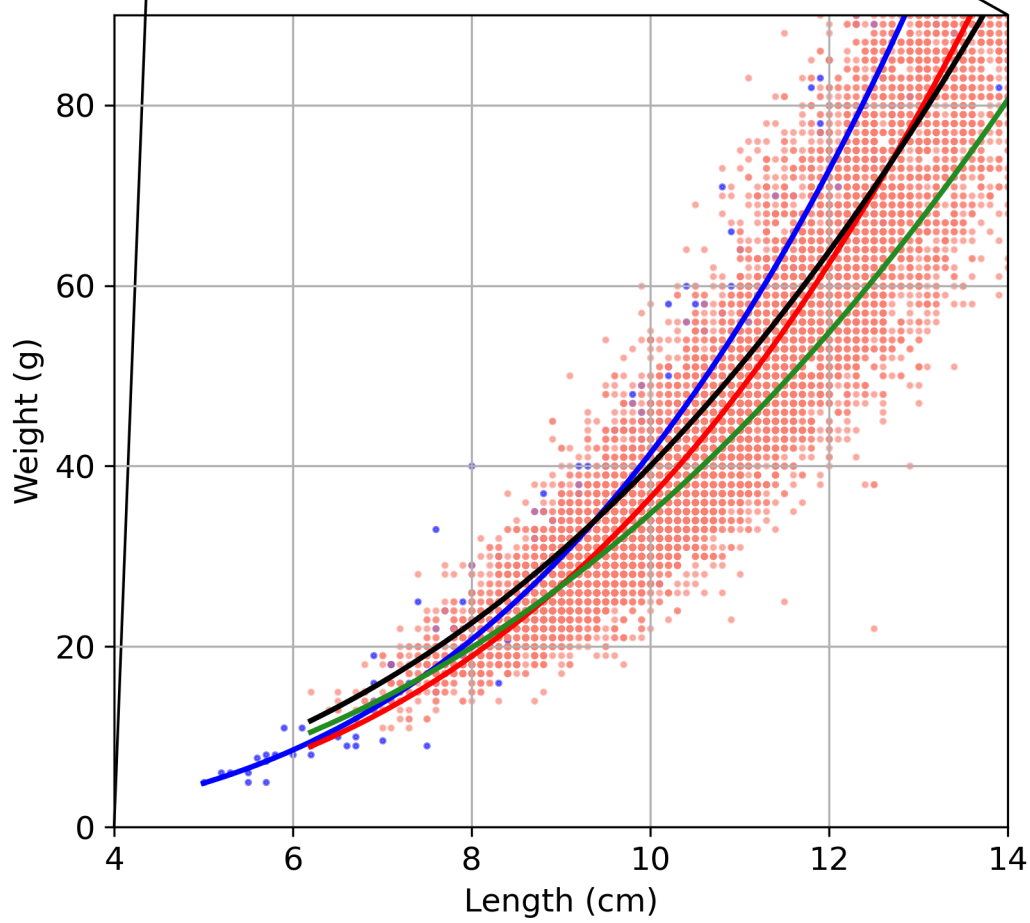

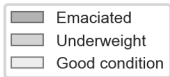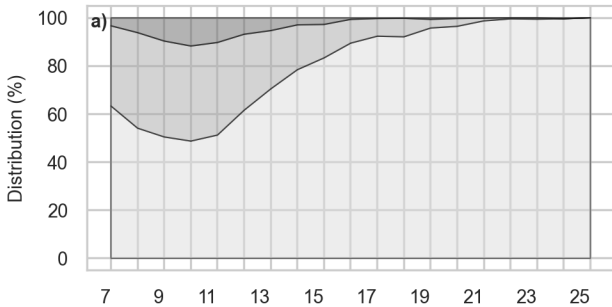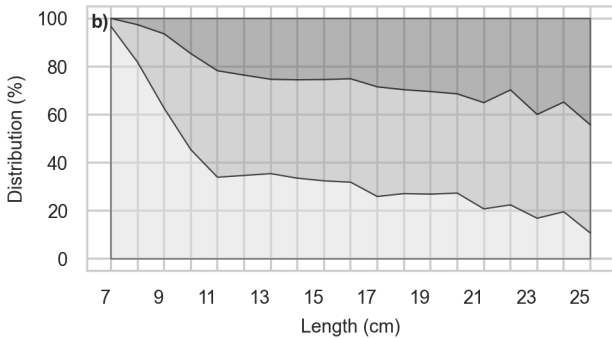

Supplement: S1 Raw images — (PDF) [file pone.0310924.s003.pdf]
